# Possible Implications of Managing Alexithymia on Quality of Life in Parkinson's Disease: A Systematic Review

**DOI:** 10.1155/2024/5551796

**Published:** 2024-08-27

**Authors:** Laura Culicetto, Caterina Formica, Viviana Lo Buono, Desirèe Latella, Giuseppa Maresca, Amelia Brigandì, Chiara Sorbera, Giuseppe Di Lorenzo, Angelo Quartarone, Silvia Marino

**Affiliations:** IRCCS Centro Neurolesi “Bonino Pulejo”, Messina, Italy

## Abstract

Alexithymia, characterized by difficulty in recognizing and verbalizing emotions, is reported to be more prevalent in subjects with Parkinson's disease (PD) than in the general population. Although it is one of the nonmotor symptoms of PD, alexithymia is often overlooked in clinical practice. The aim of this systematic review is to investigate the prevalence of alexithymia in PD, assess its impact on quality of life, and explore the rehabilitation approaches for alexithymia. Research articles, selected from PubMed, Scopus, and Web of Science, were limited to those published in English from 2013 to 2023. The search terms combined were “Alexithymia,” “Parkinson's disease,”, and “Quality of life.” Current literature review indicates that alexithymia is commonly assessed using the Toronto Alexithymia Scale (TAS-20), and it is associated with deficits in visuospatial and executive functions. Presently, rehabilitation interventions for alexithymia are scarce, and their effectiveness remains controversial. Future research should focus on developing comprehensive assessments and rehabilitation strategies for emotional processing, considering its significant impact on the quality of life of both patients and caregivers.

## 1. Introduction

Alexithymia is a personality construct characterized by difficulty in identifying and describing feelings accompanied by an externally oriented thinking style marked by a focus on external, observable events rather than internal thoughts, feelings, or fantasies. Individuals with this thinking style tend to concentrate on concrete details and practical aspects of their environment, often at the expense of introspection and emotional awareness [1, 2]. Some researchers suggest that alexithymia might be a personality characteristic that plays a role in the onset and intensity of somatic and psychological health conditions [1, 2]. Alexithymia, extensively investigated in neurological conditions [3, 4], nonneurological diseases [5, 6], and various psychiatric disorders [7, 8], is also relevant to Parkinson's disease (PD), a disorder primarily associated with motor symptoms. However, the recognition of PD's nonmotor symptoms (NMS), which significantly impact patients' quality of life, is increasing among healthcare professionals and scientists, suggesting a potential area for alexithymia research within this population. The NMS of PD encompass cognitive impairment [9, 10] and neuropsychiatric conditions like apathy [11, 12], depression [13, 14], and alexithymia [15, 16]. In addition, there are also REM behavior disorders (RBD) [17], olfactory disfunction [18], fatigue [19], sexual dysfunction [20], gastrointestinal symptoms such as dysphagia and dribbling of saliva. Further, symptoms of dysautonomia in PD may include orthostatic hypotension, bladder dysfunction, gastrointestinal dysfunction (particularly constipation) [21], and hyperhidrosis [20]. The cognitive deficits associated with PD are believed to stem from diminished dopamine activity in the nigrostriatal and mesocortical dopaminergic systems, resulting in the malfunctioning of neural networks, including the basal ganglia and related cortical areas [22]. The reduction of dopamine in key brain regions important for emotional processing, including the anterior cingulate cortex (ACC) and orbitofrontal cortex, might be a fundamental factor in the emergence of alexithymia in this condition [23, 24].

Alexithymia's features indicate deficits in both understanding and managing emotions, including challenges in using adaptive strategies for emotional regulation (like adjusting arousal levels, expressing or restraining emotions, and cognitive integration). These difficulties might also play a role in the progression and trajectory of diverse psychiatric and medical conditions, including depression and diabetes [8, 25], and can influence the effectiveness of treatment [26, 27].

Jacobs et al. [28] discovered that PD subjects showed reduced performance in tasks related to emotional facial imagery, as well as in tasks involving emotional expression and recognizing emotions in faces. Dujardin et al. [29] also found that PD subjects have difficulty interpreting facial emotions at earlier stages of the disease. Furthermore, Simons et al. [30] observed that, compared to healthy controls, PD subjects had more difficulty in posing emotional facial expressions. Studies focusing on the ability to recognize and express affective verbal expressions have similarly highlighted deficits in PD subjects regarding communication and emotions interpretation [28, 31].

In the current literature, there is variability in sociodemographic aspects such as age, sex, and education in relation with alexithymia in PD. Indeed, research has indicated that factors like age are linked to alexithymia [3, 32], while male gender may influence the expression of alexithymia [32, 33]. However, not every study has established a meaningful connection between gender and alexithymia [3], suggesting that factors beyond gender are more predictive of alexithymia [34].

Furthermore, higher levels of education appear to be inversely related to alexithymia [3, 35]. Previous studies found a link between lower education level and challenges in recognizing emotions [3], highlighting lower education as a potential risk factor for alexithymia [36, 37].

Quality of life (QoL) is a complex, multifaceted concept encompassing at least three major areas: physical, mental, and social well-being. Within the medical realm, the notion of health-related quality of life is frequently employed by researchers and clinicians. This concept particularly focuses on how an illness and/or its treatment influences a patient's self-perceived health status, as well as their subjective sense of well-being or life satisfaction [38]. In PD people, subjective elements influencing QoL encompass their perception of symptoms, physical fitness, self-image, contentment with family life, occupational satisfaction, financial circumstances, interactions with others, social support, and overall life experience. The objective factors contributing to QoL in PD include the clinical manifestations of the disease, social standing, living conditions, and the extent and intensity of social interactions [39]. QoL in PD is influenced by both motor symptoms (tremor, rigidity, bradykinesia, postural instability) and nonmotor symptoms (anxiety, sleep disturbances, autonomic dysfunctions) [40]. Disability level, daily activity dependence, and the presence of depression and cognitive decline significantly affect QoL [41]. Social factors, including socioeconomic status, living conditions, access to healthcare, and support services, as well as the quality of social interactions and support from family and friends, are also crucial [42]. In addition, the effectiveness and side effects of pharmacological interventions play a critical role in determining QoL in PD patients [43].

Given the limited research on alexithymia in PD, this review aims to explore its impact on QoL in PD population and to identify existing rehabilitation approaches for alexithymia.

## 2. Methods

### 2.1. Search Strategy

The systematic review was performed using the PRISMA (Preferred Reporting Items for Systematic Reviews and Meta-Analyses) [44] guidelines to investigate alexithymia in PD, focusing on the impact on QoL and rehabilitation approaches for alexithymia. This systematic review did not involve human nor animal data collection. Therefore, ethical approval was not required.

Studies published from 2013 to 2023 were identified by searching on PubMed, Web of Science, and Scopus. The search combined the following terms (“affective symptoms”[MeSH Terms] OR (“affective”[All Fields] AND “symptoms”[All Fields]) OR “affective symptoms”[All Fields] OR “alexithymia”[All Fields] OR “alexithymia s”[All Fields]) AND (“parkinson disease”[MeSH Terms] OR (“parkinson”[All Fields] AND “disease”[All Fields]) OR “parkinson disease”[All Fields]) AND (“quality of life”[MeSH Terms] OR (“quality”[All Fields] AND “life”[All Fields]) OR “quality of life”[All Fields]).

### 2.2. Study Selection

After the removal of the duplicates, all the articles were evaluated based on the titles and abstracts by three of the researchers (LC, VLB, and CF), independently. These researchers read the full-text articles deemed suitable for the study, and performed data collection to reduce the risk of bias. Any disagreement was discussed by the three above authors.

The inclusion criteria were (a) subjects with PD and alexithymia and (b) PD subjects treated and not treated with deep brain stimulation. The exclusion criteria were (a) atypical Parkinsonism and (b) article not written in English.

**Figure 1:**
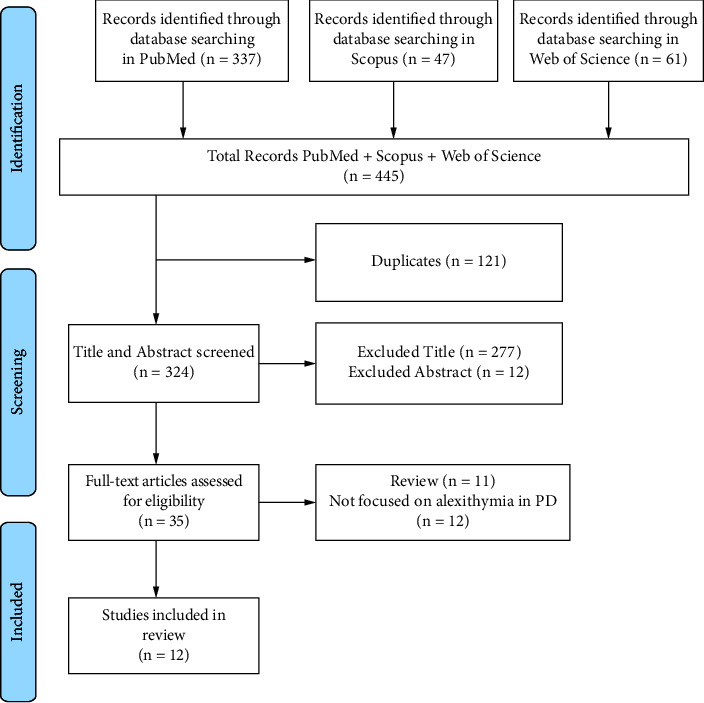
PRISMA flowchart showing identification and inclusion of studies in the systematic review.

Electronic searches identified 445 papers. We rejected 121 studies because they were duplicates. After reading the full text of the selected publications and applying the predefined inclusion criteria, we excluded 277 articles based on title and 12 on abstract; 12 because they did not focus on alexithymia in PD and 11 because they were review. After an accurate revision of full manuscripts, 12 articles satisfied the inclusion/exclusion criteria (Figure 1).

### 2.3. Data Extraction

One individual (LC) extracted all relevant data from the included studies. General information included (first author's name and year), study design, study population characteristics (population size, age, gender, duration of Parkinson's disease, and Hoehn and Yahr stage), study interventions (drug and dosage), endpoint UPDRS total score, and UPDRS Part II and III subscores. A second individual (CF) double-checked the extracted data. Conflicts were resolved by discussion or by a third reviewer when needed.

## 3. Results

The exploration of the relationship between alexithymia and PD has gained significant attention in recent research (Table 1). Based on the reviewed studies, the prevalence of alexithymia in PD varies considerably across different cohorts. Sengul et al. [45] reported a prevalence of 35% for alexithymia, with an additional 33% of patients classified as borderline alexithymic. Similarly, Alvarado-Bolaños et al. [46] found a higher prevalence rate of 56% in their PD sample. Goerlich Dobre [47] reported that 18% had clinical alexithymia, and 26% had moderate alexithymia. The distribution of alexithymia across age and gender shows that male patients are more frequently affected. Kenangil et al. [45] reported a higher prevalence of alexithymia in males compared to females within their cohort, where the mean age was 60.33 years. Sengul et al. [48] also observed a mean age of 71.2 years with a higher proportion of male patients (62.8%). In terms of disease stage, Kenangil et al. [45] identified significant differences in Hoehn and Yahr (HY) stages between alexithymic and nonalexithymic PD patients, with higher HY stages correlating with increased alexithymia prevalence. Alvarado-Bolaños et al. [46] supported this finding, indicating that alexithymic patients were more likely to be in advanced HY stages. The study by Bogdanova et al. [49] found a significant correlation between alexithymia ratings and disease stage, indicating that higher disease stages are associated with higher alexithymia levels. On the contrary, Sengul et al. [48] did not find a significant correlation between H&Y stages and alexithymia.

The prevalence and characteristics of alexithymia in PD vary across different stages and treatment modalities. Alvarado-Bolaños et al. [50] reported that alexithymia in early PD patients treated pharmacologically is associated with both motor and nonmotor symptoms. However, they did not provide a detailed analysis of how specific pharmacological treatments influence alexithymia, highlighting the need for further investigation. In patients with moderate PD and motor and nonmotor fluctuations, the literature lacks detailed information on the characteristics and frequency of alexithymia, underscoring the necessity for future studies to differentiate between these subpopulations. Castelli et al. [51] observed that Deep Brain Stimulation (DBS) did not significantly alter alexithymia levels compared to pre-DBS patients. This suggests that alexithymic symptoms may persist in advanced PD stages, irrespective of surgical interventions, pointing to the need for additional therapeutic strategies. Alvarado-Bolaños et al. [50] also emphasized that longer disease duration and more severe motor symptoms are associated with higher levels of alexithymia. This implies that as PD progresses, the severity of alexithymic symptoms increases. In contrast, Goerlich-Dobre et al. [52] found no significant correlation between alexithymia scores and disease duration or dopaminergic medication dosage, such as total levodopa equivalent daily dose (LEDD) or dopamine agonist LEDD. These conflicting findings highlight the need for further research to clarify the relationship between disease duration, symptom onset, and alexithymia.

Klietz et al. [53] focused on the impact of alexithymia on both people with Parkinson (PwP) and their caregivers. Employing disease-specific questionnaires, they discovered a notable correlation between alexithymia in PwP and reduced health-related quality of life (HR-QoL), with specific aspects like difficulty in identifying and describing feelings being particularly influential. Caregiver burden was also significantly associated with alexithymia in PwP, particularly with aspects of alexithymia like external-oriented thinking and identifying feelings.

Furthermore, Castelli et al. [51] investigated the impact of DBS on alexithymia in PD subjects. They compared groups undergoing STN-DBS with those who did not, finding no significant difference in alexithymia levels post-DBS, though both groups exhibited higher alexithymia than controls. No significant correlation was found between alexithymia and the severity of Parkinson's disease. In a related longitudinal study, Dafsari et al. [49] reported a significant decrease in alexithymia prevalence and improvements in QoL and motor symptoms following STN-DBS with a significant reduction in the levodopa-equivalent daily dose (LEDD).

Further exploring the broader implications of alexithymia in PD, Gul et al. [46] linked high levels of alexithymia with cognitive decline in PD patients, identifying alexithymia as a significant predictor of cognitive performance. This was supported by Bogdavona et al. [48], who found a correlation between alexithymia and nonverbal cognitive tasks, suggesting a deeper connection with certain cognitive functions. Notably, the severity of alexithymia, but not apathy or depression, correlated with the Hoehn and Yahr index of PD disease stage.

The recent study by Alvarado-Bolanos et al. [50] aimed to identify determinants of alexithymia in PwP, linking it with factors like education level and urinary symptoms. They also emphasized the impact of alexithymia on the QoL in PD.

Sengul et al. [45] associated alexithymia with poorer performance in visuospatial and executive function tests and depressive symptoms, highlighting the complex interplay between alexithymia and cognitive dysfunctions in PD. Kenangil et al. [54] added to this by noting a correlation between the F3 subscale of Toronto Alexithymia Scale (TAS-20) scores and Montreal Cognitive Assessment (MOCA-TR) scores, indicating a link between alexithymia and cognitive functions. There was no significant difference in depression levels (BDI scores) between PD subjects and controls. The study did not find a relationship between alexithymia and the duration of PD or total levodopa dose.

The impact of PD on emotional processing was further detailed by Enrici et al. [47], who noted significant deficits in emotion recognition, representation, and regulation in PD population, particularly in interpreting facial expressions and social cues. This impairment was distinct from any general cognitive of psychiatric status, including depression, anxiety, or apathy.

Lastly, Goerlich-Dobre [52] and Sonkaya et al. [55] provided unique perspectives. Goerlich-Dobre et al. [52] showed that alexithymia, especially difficulties in identifying and describing feelings, is linked with impulse control disorders in PD. Sonkaya et al. [55] emphasized the relevance of alexithymia as a nonmotor symptom in PD, correlating it with the severity of motor symptoms.

## 4. Discussion

### 4.1. Alexithymia and Comorbidity in PD: The Impact on Quality of Life

In this systematic review, we explored the impact of alexithymia on the QoL of people with PD. The distress/protection model defines QoL as the overall balance between protective and distressing factors [56] where the prevalence of distressing factors (such as severe depressive symptoms) over protective factors (such as family social support) results in lower QoL. The presence of both motor and psychosocial dysfunctions and psychiatric comorbidities in PD patients can lead to a decrease in their QoL [57–61].

The impact of NMS on HRQoL has been examined in some longitudinal studies, showing that the nonmotor symptoms significantly increase over time affecting the quality of life [61–63]. This might apply specifically to the so-called honeymoon period, when patients are adequately pharmacologically compensated from the motor perspective [49, 62, 64, 65].

Numerous studies increasingly focus on NMS as outcome measures, but they do not always assess their impact on HRQoL and caregivers' burden [66, 67].

A single study assessed the QoL of caregivers of Parkinson people using the Parkinson's disease caregiver burden questionnaire (PDCB) [53]. This evaluation is crucial because neuropsychiatric symptoms can disrupt familial and social dynamics, leading to nursing home placement, and having severe negative effects on patients' HRQoL and caregivers' burden [65]. According to previous findings, the studies included in this systematic review suggest that NMS, particularly alexithymia, are significant contributors to HRQOL [62, 68].

Alexithymia, characterized by difficulties in identifying and expressing emotions, is a notable nonmotor symptom in PD. The reviewed studies suggest a link between alexithymia and cognitive dysfunction in PD patients, particularly in the domains of visuospatial and executive functions, indicative of frontostriatal and parietal dysfunction in PD [48].

Specifically, the alexithymia aspect “externally oriented thinking,” measured by the F3 subscale of the TAS-20, correlates with the visuospatial domain of the MOCA [54]. Bogdanova et al. [48] discovered that higher TAS-20 scores are linked to diminished performance in visual-spatial ability tests, involving emotional stimuli, suggesting a link between alexithymia and right hemisphere dysfunction, as the right hemisphere plays a key role in processing visuospatial information. Furthermore, Sengul's study [45] showed that patients with elevated levels of alexithymia exhibit lower performance in the clock drawing test, a measure of compromised visuospatial function.

Interestingly, alexithymia in PD appears independent of motor neurological symptoms, dopaminergic treatment, and the laterality and type of motor symptom onset [54]. This suggests that alexithymia may arise from different underlying mechanisms than those driving the motor symptoms of PD. A few studies have investigated the link between motor symptoms and alexithymia. In the study by Alvarado-Bolanos [50], a correlation was observed between the intensity of motor symptoms and the severity of alexithymia. Costa et al. [69] noted that the severity of motor symptoms was similar between alexithymic and nonalexithymic people with PD, although the postural instability and gait disorder subtype is linked with greater challenges in recognizing and articulating emotions.

In Addition, Enrici et al. [47] reported that there was no significant association between the use of dopamine medication and the presence of alexithymia in people with PD. Similarly, Goerlich-Dobre et al. [52] found no relationship between alexithymia and various factors such as disease duration, total levodopa dose, or dopamine agonists' dosage, suggesting that the use of dopaminergic medications and the progression of neurodegenerative processes in PD do not appear to negatively impact patients' emotional self-awareness. Conversely, some studies have suggested A potential link between alexithymia and PD severity. Sonkaya et al. [55] examined the relationship between alexithymia and Parkinson's disease severity, finding that higher levels of alexithymia were associated with UPDRS III scores above 30. However, Castelli et al. [51] did not observe a significant connection between alexithymia and disease severity.

The relationship between alexithymia and depression in PD is complex and somewhat conflicting. While some studies have found a significant correlation between these two conditions [3, 70], others suggest that aspects of alexithymia may be independent of depression [54] This underscores the multifaceted nature of neuropsychiatric symptoms in PD and their impact on patients' quality of life.

Apathy is another crucial NMS to address in PD due to its substantial impact on QoL and patient disability [71, 72]. In the study conducted by Bogdanova et al. [48], apathy levels, but not depression, were linked to alexithymia severity. Both alexithymia and apathy are conditions that can be treated, and early detection and intervention might safeguard the QoL and lessen disability in individuals with PD. Furthermore, subjects who have difficulty recognizing and understanding their own emotions, and who often suppress their feelings as a way of managing them, may be vulnerable to being overwhelmed by negative emotions that they cannot identify or articulate. In this regard, findings reveal that not only clinical levels of alexithymia but also moderate levels are significantly correlated with a higher incidence of impulse control disorders (ICDs) in PD [52].

The evaluation of specific treatment strategies to improve the NMS of Parkinson's disease and, in turn, HRQoL has been limited by few studies. Improvement in NMS was demonstrated by infusional therapies, such as subcutaneous apomorphine and intrajejunal levodopa, but there was no specific investigation of the impact on HRQoL [73, 74].

### 4.2. The Assessment and Rehabilitation of Alexithymia

A significant methodological concern in PD research is the assessment of alexithymia. Among the analyzed studies, only one employed Bermond-Vorst Alexithymia Questionnaire (BVAQ) [46], a reliable and valid assessment tool including 40 statements rated on a 5-point Likert scale. This questionnaire encompasses five subscales that cover both cognitive and affective aspects of alexithymia. The subscales of fantasizing and emotionalizing concern the affective dimension, while identifying, analyzing, and describing subscales represent the cognitive dimension. The total score can range from 40 to 200, with higher scores indicating greater levels of alexithymia [75, 76]. The internal consistency for the total BVAQ score and certain subscales (Verbalizing, Identifying) is acceptable (Cronbach's alpha around 0.70–0.80). However, some subscales (Fantasizing, Emotionalizing) exhibit lower internal consistency [76]. Most studies have employed the TAS-20 [77], a self-report questionnaire designed to measure alexithymia's three primary aspects: difficulty in identifying and describing feelings, and a tendency towards externally oriented thinking. Participants rate their agreement with 20 statements on a 5-point Likert scale. The TAS-20, widely adopted in both clinical and nonclinical populations, is considered to have strong convergent and discriminant validity [77]. The reliability of the total scale equals 0.81, and the reliabilities of the three factors are 0.78, 0.75, and 0.66 (F1, F2, F3, respectively) [77]. In addition, the TAS-20 has shown good test-retest reliability. However, its psychometric properties specific to PD subjects remain unexplored, presenting a potential limitation for two key reasons. Firstly, accurately responding to the TAS-20 requires meta-cognitive abilities, which might be compromised in PD subjects due to possible reductions in executive function efficiency. Indeed, executive performance has been shown to correlate with TAS-20 scores [48].

Secondly, completing the questionnaire necessitates intact self-awareness. This challenge extends to all self-report assessments that require individuals to evaluate and recognize their cognitive and emotional traits. Prigatano and Schacter [78] describe self-awareness as the ability to perceive oneself objectively while maintaining subjectivity. Impairments in self-awareness could therefore influence TAS-20 responses. Recent studies indicate that self-awareness may be diminished in PD subjects. For instance, Kudlicka et al. [79] observed that PD people with executive dysfunctions had more difficulty acknowledging their cognitive impairments compared to those without such dysfunctions. Another study involving PD subjects by Amanzio et al. [80] found a link between awareness of movement disorders and dopamine stimulation. Specifically, they noted that PD subjects in the “on state” showed reduced awareness of dyskinesia-related motor deficits, which correlates with executive functions, whereas in the “off state,” their awareness of hypokinesia remained intact. Therefore, a clear and precise operational definition of alexithymia in PD is essential for ensuring consistency and accuracy in research. Alexithymia is commonly characterized by difficulties in identifying and describing feelings, a limited imaginal capacity, and an externally oriented cognitive style [1, 2]. In the context of PD, these traits may be compounded by the neurological and psychological challenges inherent to the disease. The prevalence of alexithymia in PD could potentially vary depending on the screening tool used. The TAS-20 provides a broad measure, while a dedicated alexithymia scale tailored to the specific neurological and psychological context of PD might reveal different prevalence rates. This underscores the need for ongoing research to develop and validate such specialized tools.

In the current landscape of research on PD, there is a notable scarcity of comprehensive studies focusing on both pharmacological and nonpharmacological treatments for alexithymia in PD. Despite the recognized prevalence of alexithymia and its detrimental impact on QoL, there remains a gap in the literature regarding specific interventions aimed at ameliorating this condition.

The effectiveness of Subthalamic Nucleus Deep Brain Stimulation (STN-DBS) in treating alexithymia in PD subjects has yielded mixed results. Dafsari et al. [49] contribute significantly to this domain by showing that bilateral STN-DBS can positively influence alexithymia, as evidenced by significant improvements in alexithymia scores and reduced prevalence. These benefits are closely linked to enhanced QoL outcomes. However, Dafsari et al. [49] also emphasize the necessity for further research to understand the enduring impacts of DBS on alexithymia. Contrastingly, Klietz et al. [53] reported only marginal benefits from STN-DBS in alleviating alexithymia symptoms. This study introduces the concept that mindfulness-based interventions could be advantageous for PD subjects. By promoting heightened awareness of feelings and emotions, these interventions might also contribute to an improved HRQoL. Further complicating the picture, Castelli's study suggests a neutral effect of STN-DBS on alexithymia in PD, neither significantly improving nor exacerbating it. The persistently high prevalence of alexithymia among both DBS and pre-DBS groups underscores the need for an integrative approach to PD management that includes psychological components. This is particularly relevant considering the association between STN-DBS and increased risks of suicide [81], and the higher prevalence of suicidal thoughts among individuals with alexithymia [82].

In populations without PD, mindfulness practices have shown potential in modifying alexithymic tendencies [83]. While alexithymia often poses a challenge to successful psychotherapy, mindfulness-based methods have shown efficacy in ameliorating this condition. These approaches could be advantageous for PD subjects and their caregivers, potentially enhancing their HRQoL. However, the effectiveness of such interventions in PD people might be limited due to attentional cognitive deficits typical in this population. Therefore, established programs like mindfulness-based stress reduction might offer valuable insights into the effects of mindfulness on alexithymia in PD. Klietz et al. [53] also highlight the significant role of mindfulness in caregivers, underscoring its potential to alleviate caregiver burden.

The presence of alexithymia in PD people has implications for their care [53]. Understanding and addressing this condition could lead to better management strategies, potentially improving the QoL of this population. This involves considering the broader neuropsychiatric context of PD, including cognitive and emotional aspects, beyond the primary motor symptoms.

In summary, the studies reviewed collectively underscore the multifaceted impact of alexithymia on PD patients, influencing aspects from QoL and cognitive functions to emotional processing and impulse control. A notable limitation in these studies is the absence of control groups. In addition, there is a pressing need to standardize alexithymia rehabilitation approaches to improve patient's quality of life.

## 5. Limitations

The main limitation of the present review is that the process of selection and recruitment of PD patients varied across the studies selected, raising concerns about the representativeness of the samples. In terms of cognitive performance, although many studies required Mini Mental State Examination (MMSE) scores of >26 or 28 for inclusion [47, 48], some studies report that alexithymic PD patients often exhibit lower scores on cognitive assessments. For instance, Alvarado-Bolaños et al. [50] observed that alexithymic PD patients scored lower on the MoCA by an average of 1.7 points compared to nonalexithymic PD patients, suggesting that cognitive impairment is more pronounced in the alexithymic group. In addition, Bogdanova et al. [48] found that alexithymia in PD patients was significantly correlated with poorer performance on tasks requiring executive and visuospatial processing, such as the Trail Making Test (TMT) and Raven's Coloured Progressive Matrices (RCPM). These findings underscore the importance of considering cognitive impairment when assessing alexithymia in PD patients.

Moreover, many studies had small sample sizes, limiting the ability to explore subgroup effects, such as differences by motor symptom onset (tremor vs. nontremor) or interactions with gender [46, 54]. Furthermore, there is no standardized operational definition of alexithymia in PD, leading to potential underdiagnosis due to the lack of objective diagnostic criteria. This variability makes it difficult to compare findings across studies and to draw firm conclusions. The role of comorbid conditions such as stroke, cerebrovascular disease, Alzheimer's disease, and PD-dementia has not been adequately evaluated. In addition, many studies did not adequately address the potential impact of pharmacotherapy, including antidepressants and dopaminergic medications, on psychological variables [45, 51, 53]. This omission could lead to incomplete or biased interpretations of the data.

Longitudinal studies are needed to better understand the temporal relationship between alexithymia and PD, and whether alexithymia could be a preceding nonmotor symptom (NMS) similar to sleep behavior disorder or olfactory dysfunction [54]. Future research should include neuroimaging studies to determine the neuroradiological correlates of alexithymia and focus on its treatment. These studies can provide deeper insights into the brain regions involved and potential therapeutic targets.

## 6. Conclusion

There is a growing body of evidence suggesting that NMS of PD have a negative impact on HRQoL. However, there is still a small number of therapeutic clinical trials that deal with nonmotor aspects of PD, particularly alexithymia, and their effects on HRQoL. Alexithymia in PD is reported to be higher than in the general population, and it is associated with cognitive impairments, particularly in visuospatial and executive functions and affective disorders such as depression, apathy, and impulse control disorders. The clinical implication of these findings is that the best assessment and management of alexithymia could improve the QoL of patients and reduce the caregiver burden. Despite the recognized prevalence of alexithymia and its negative impact on QoL, there is a gap of literature on specific interventions aimed at ameliorating this condition. Further research is required to establish the best rehabilitation approaches for enhancing patient well-being.

## Figures and Tables

**Table 1:** The main studies concerning alexithymia in Parkinson's disease.

Authors	PD population	Disease duration H&Y	Education	Cognitive and affective tests	Control group	Rehabilitation
Klietz et al. 2020	119 (45 females), average age 68.7; 119 caregivers (78 females), average age 65.4	Mean disease duration 12.0. H&Y stage mean 2.9	Na	TAS-26; SF-36; BDI; MDS-UPDRS part II	Na	Na
Castelli et al. 2014	DBS group: 27 (11 females), average age 61.4, pre-DBS group: 38 (15 females), average age 59	DBS group: Severity of the disease 47.3; pre-DBS group: 44.3	DBS group: average 9.5, pre-DBS group: average 9, control group: average 11.2	TAS-20; BDI; MMSE; PM-47; BWR; PAL; TMT; MCST; FAB	27 HC (12 females), mean age 60.1	DBS
Gul et al. 2019	60 (30 females), mean age 60.28	Disease duration 5.06	Na	BVAQ; MOCA	60 HC (30 females), mean age 60.33	Na
Dafsari et al. 2019	39 (14 females), mean age 62.8	Disease duration 9.6 H&Y 2.5	Na	TAS-20; PDSS; UPDRS-II; AES; HADS; NMSQ; SRMI	Na	Bilateral STN-DBS
Alvarado- Bolanos et al. 2020	98 (44 females), mean age 62.7	Mean disease duration 10. 70.4% of patients had mild disease (H&Y Stage 1 or 2), 20.4% had moderate disease (H&Y Stage 2), and 9.2% had severe disease (H&Y Stage 4 or 5)	Mean education level 10.0	MDS-UPDRS; NMSS; MoCA; TAS-20	98 HC (57 females), mean age 61.5	Na
Bogdanova et al. 2013	22 (12 women), mean age 61.8	Duration of disease 7.8. H&Y S2 (1–3)	15.6	TAS-20; BDI-II; FAS; TMT; RCPM; BVSQB; clock reading test; digit span	22 HC (12 females), mean age 61.0, education 16.6	Na
Sengul et al. 2020	35 (13 females), mean age 71.2	Mean duration 6.5. H&Y 1.5	Illiterate, *n* 2 (5.7%), literate, *n* 4 (11.4%) Primary school, *n* 27 (77.1%), high school, *n* 2 (5.7%)	TAS-20; GDS; WMS-R; WAIS Stroop test; logical memory; JoLO; BFRT; BNT; verbal fluency tests; CDT	Na	Na
Enrici et al. 2015	32 (15 females), mean age 57.97	H&Y Off 2.81; H&Y On 1.60	9.47	TAS-20; MMSE; FAB; STAI-X1-2; BDI; Attentional Matrices task; PM; BWR; Corsi's block- tapping test; TMT; MCST; AES	25 HC (14 females), mean age 56.32, education 10.80	Na
Goerlich-Dobre et al. 2014	91 (27 females), mean age 62.3	Mean duration 8.5	Na	TAS-20; ERQ; BIS-11; BIS/BAS; BDI- II; BAI; QUIP- RS	Na	Na
Kenangil et al. 2020	43 (18 females), mean age 60.33	Duration of disease 5.50; H&Y 1.66	Na	TAS-20; MOCA; BDI	40 HC (19 females), mean age 59.25	Na
Sonkaya et al. 2019	67 (27 females), mean age 61.20	Duration of illness 9.56	9.14	TAS-20; BDI; MMSE; UPDRS III	70 HC (32 females), mean age 58.32	Na

UPDRS-III = Unified Parkinson's Disease Rating Scale; PDSS = Parkinson's Disease Sleep Scale-1; TAS-20 = 20-item Toronto Alexithymia Scale; BVAQ = Bermond-Vorst Alexithymia Questionnaire; GDI = Geriatric Depression Inventory; MOCA = Montreal Cognitive Assessment; ERQ = Emotion Regulation Questionnaire; BIS-11 = Barratt Impulsiveness Scale; BIS/BAS = Behavioral Inhibition/Approach Scale; BDI-II = Beck's Depression Inventory; BAI = Beck's Anxiety Inventory; QUIP-RS = Questionnaire for Impulsive-Compulsive Disorders in Parkinson's Disease Rating Scale; NA: not available; SF-36 = 36 Health Survey Quality of Life Questionnaire Short Form; GDS = Geriatric Depression Scale; PDCB = Parkinson's Disease Caregiver Burden Inventory; MDS-UPDRS = Movement Disorders Society Unified Parkinson's Disease Rating Scale; PD = Parkinson's disease; TAS-26 = Toronto Alexithymia Scale 26 (F1: difficulties identifying feelings, F2: difficulties describing feelings, and F3: external oriented thinking); AES = Apathy Evaluation Scale; HADS = Hospital Anxiety and Depression Scale (A, anxiety; D, depression subscale); NMSQ = Nonmotor Symptoms Questionnaire; NMSS = Nonmotor Symptom Scale total score; PDQ-8 SI = Parkinson's Disease Questionnaire-8 Summary Index; SRMI = Self-Report Manic Inventory; FAS = Controlled Oral Word Association Test; TMT = Trail Making Test; RCPM = Raven's Coloured Progressive Matrices; BVSQB = Boston Visuo-spatial Quantitative Battery; MCST = Nelson Modified Card Sorting Test; BWR = bisyllabic word repetition; PAL = paired-associate learning; HR-QoL = health-related quality of life; STN-DBS = deep brain stimulation of the subthalamic nucleus; ICDs = impulsive-compulsive disorders. H&YS = Hoehn and Yahr score; FAB = frontal assessment battery; WMS-R = Wechsler Memory Scale Revised; PDCB = Parkinson's disease caregiver burden questionnaire; CDT = clock drawing test, BNT = Boston naming test; BFRT = Benton face recognition test; JoLO = Benton jugdment of line orientation; HC = healthy controls; PwP = people with Parkinson's disease.
